# Model Simplification of Deep Random Forest for Real-Time Applications of Various Sensor Data

**DOI:** 10.3390/s21093004

**Published:** 2021-04-25

**Authors:** Sangwon Kim, Byoung-Chul Ko, Jaeyeal Nam

**Affiliations:** Department of Computer Engineering, Keimyung University, Daegu 42601, Korea; swkim@stu.kmu.ac.kr (S.K.); niceko@kmu.ac.kr (B.-C.K.)

**Keywords:** model simplification, deep random forest, rule elimination, transparent machine learning, interpretable machine learning

## Abstract

The deep random forest (DRF) has recently gained new attention in deep learning because it has a high performance similar to that of a deep neural network (DNN) and does not rely on a backpropagation. However, it connects a large number of decision trees to multiple layers, thereby making analysis difficult. This paper proposes a new method for simplifying a black-box model of a DRF using a proposed rule elimination. For this, we consider quantifying the feature contributions and frequency of the fully trained DRF in the form of a decision rule set. The feature contributions provide a basis for determining how features affect the decision process in a rule set. Model simplification is achieved by eliminating unnecessary rules by measuring the feature contributions. Consequently, the simplified and transparent DRF has fewer parameters and rules than before. The proposed method was successfully applied to various DRF models and benchmark sensor datasets while maintaining a robust performance despite the elimination of a large number of rules. A comparison with state-of-the-art compressed DNNs also showed the proposed model simplification’s higher parameter compression and memory efficiency with a similar classification accuracy.

## 1. Introduction

In the field of artificial intelligence (AI), the development of deep neural networks (DNN) has been a remarkable success, surpassing the achievements of AI from the last 60 years. Although the structures of recent DNNs continue to deepen and widen, resulting in improved recognition rates, several challenges remain: (1) when a DNN encounters a scenario that differs from the scenario used during the training phase, an instability occurs in that the structure cannot be modified based on the scenario, (2) a DNN is programmed on the basis of a small amount of knowledge and is superficial in that it does not have common sense regarding the world and human psychology [1], (3) recent DNN models continue to become wider and deeper to achieve a better performance, and may not be suitable for a variety of applications with limited memory or computational times, (4) a DNN system is greedy because it requires numerous training data, and (5) because the output of a DNN is calculated through a black box, it cannot be accurately understandable.

The first and second issues require more research to reduce the structural gap between a DNN and the actual human brain in terms of neuroscience, whereas the remaining issues can be solved by changing the current structure of the DNN. To reduce the size of the DNN network (issue 3), some studies have focused on compressing a DNN with a similar performance as the original models while reducing the size and width of the DNN network, e.g., using a knowledge distillation [2,3], transferred/compact convolutional filters [4], low-rank factorization, and parameter tuning and sharing [5]. However, a compressed DNN model still requires a large number of parameters and a large amount of memory for processing the resources required for multiplication [6]. In addition, to create a deep model that can be trained using a small number of training data (issue 4) without a backpropagation, new approaches have been attempted for linking random forests (RFs) [7,8] or random ferns [6] to layers instead of neurons in a deep model. These deep random classifier-based models link several ensemble algorithms to multiple layers with non-differentiable components and do not use backpropagation during training.

Recently, studies on interpretable machine learning (IML) have been actively conducted to improve the limitations of a black box model regarding the learning process (issue 5 above), which is an issue of deep learning. IML is also referred to as eXplainable artificial intelligence (XAI) interchangeably, but the purpose of this paper is to reduce the size of the model to provide as much transparency as possible, so IML terminology is used in this paper. IML is a technology that allows humans to understand and correctly interpret the behavior and the end result of an ML model to explain how the result is generated [9]. Therefore, unlike a black-box model, users can check whether the decision made by an AI model is the best decision before making the final determination through a white-box model.

Studies on the development and testing of an IML learning model have been conducted to improve transparency while maintaining a high-level learning ability by modifying the existing machine learning technologies or developing new ones. The technical approach for IML can be divided into the following: (a) explaining a decision of the learning model (ELM) [10,11,12,13,14] and (b) interpreting the learning model (ILM) [15,16,17,18,19,20,21,22,23].

The ELM is also called a post hoc explanation because it tries to explain why a black-box model behaved that way. The ELM does not have full control over the model’s structure, and focuses on explaining the model’s decision by identifying the relevant input variable. Interpretation techniques are applied to the output of nonlinear machine learning models to produce a heat map (e.g., a highlighted image or text) of the interpretable input variables [13]. The ELM method gives users an extremely intuitive result by emphasizing the input variables through a prediction and redistribution of the learning model to determine which part of the input feature influenced the outcome. However, because ELM-based methods still depend on a complex black-box model, it is difficult to explain what rules are used in the deep network to actually reach such a decision.

Unlike ELM, the ILM method called transparent design tries to reveal how a model functions. ILM aims to produce models that are inherently interpretable in a different way from a black box-based ELM approach. The representative model of an ILM is a rule-based algorithm, such as stochastic AND/OR graphs (AOGs), decision lists, and decision trees, because users can easily understand simple rules [15].

In this study we focus on the development of a new ILM-based model simplification method instead of an ELM-based approach, which can maintain the important properties of the model structure and redefine the rules without sacrificing the performance. An early version of this paper was preprinted on TechRxiv (Kim et al., 2020) [16]. In this paper, we performed additional experiments analyzing the performance to prove model simplification. Unlike an ELM-based approach that focuses on a heat map of the input variables when using a DNN, we wish to understand why particular decisions were made and generate models explaining such decisions while maintaining the same predictive performance.

The remainder of this paper is organized as follows. In Section 2, we present an overview of the related studies on model simplification. We present the details of our proposed method in terms of the feature contribution and rule elimination in Section 3. Section 4 provides a comprehensive evaluation of the proposed method based on the results of various experiments. Finally, in Section 5, we provide some concluding remarks and future works.

## 2. Related Studies

As described in the Introduction section, the purpose of this study is to propose a new deep model simplification algorithm based on ILM using a deep random forest (DRF) that shows performance similar to that of a DNN but does not rely on a backpropagation. Therefore, this section introduces the related research focusing on an ILM-based model simplification and DRF. 

### 2.1. ILM Based Model Simplification

An AOG [17] generates an AND–OR relationship graph of the characteristics of the input data (e.g., sketch, color, texture, and position of an object in an image) and confirms the classification based on the node connected to the classification result. Liu et al. [18] proposed a rule-based regression algorithm that uses one-norm regularized RFs. This approach simultaneously extracts a small number of rules from the generated RF and eliminates unimportant features. However, if the rules of the trees are excessively reduced to increase the analysis capability of the model, an issue occurs in that the performance is significantly reduced.

Bayesian rule lists (BRLs) [19] are based on a decision tree as a preliminary interpretable model providing a concise and convincing capability to gain the trust of domain experts. A BRL employs a prior structure to encourage sparsity and yield a posterior distribution over the possible decision lists. Lakkaraju et al. [20] proposed interpretable decision sets, which are sets of independent if–then rules, and a framework for building predictive models that are highly accurate and yet highly interpretable. As each rule can be applied independently, decision sets are simple, concise, and easily interpretable. A scalable Bayesian rule list (SBRL) [21] was proposed as a faster variant of a BRL. An SBRL is used to build probabilistic rule lists that are two orders of magnitude faster than the previous BRL. Rule list algorithms are competitors to decision tree algorithms and are associative classifiers in that they are built from pre-mined association rules. However, such methods have an issue in that their performance is significantly degraded when excessively reducing the rules of the tree to improve the interpreting power of the model.

By contrast, other researches have tried to improve interpretability by changing the structure of the neural networks (NN). Yang et al. [22] proposed the use of an explainable NN (xNN) subject to interpretability constraints in terms of the additivity, sparsity, orthogonality, and smoothness. A complex function is decomposed into sparse additive subnetworks and the projection indexes are forced to be mutually orthogonal such that the resulting subnetworks tend to be less confounded with each other. However, a NN-based method still depends on the backpropagation, which requires the use of a black box model during the learning process. In addition, in terms of transparency in a machine learning approach, the choice of hyperparameters such as the learning rate and batch size has a more heuristic, non-transparent algorithmic nature [23].

### 2.2. Deep Random Models

Apart from the high recognition rate of a DNN, certain limitations such as an overly large number of hyper-parameters requiring parameter tuning, a black-box model created through a gradient backpropagation, high processing costs, and the amount of training data are a significant burden to explain a DNN [24]. As an alternative approach, a deep ensemble classifier consisting of several RFs or ferns has been researched.

A multi-grained cascade forest called gcForest [8] was the initial trial to generate a deep forest ensemble with a cascade structure. To avoid a gradient backpropagation, the cascade levels are adaptively determined using an *K*-fold cross-validation, which provides a performance similar to that of a DNN, although it was trained using only a small amount of data.

A forward thinking deep random forest (FTDRF) [7] replaces the neurons of deep neural nets with decision trees instead of RFs. Input data are mapped forward through the layers to create a new learning problem. This process is repeated to convert the data of a single layer into multiple layers at a time.

Multi-layered gradient-boosting decision trees (mGBDTs) [25] build blocks for each layer with an explicit emphasis on representation learning to learn hierarchical distributed representations through the stacking of several layers of a regression GBDT.

As the application of a DRF, a Siamese deep forest [26] was proposed. This method defines the class distributions in a deep forest as the weighted sum of the tree class probabilities such that the weights are determined to reduce the distances between similar pairs of images and increase them between dissimilar points.

The lightweight multi-layered random forest (LMRF) model [24] consists of a layer-to-layer RF. Each neuron of a DNN layer is replaced with an RF, and each layer consists of several types of RFs. Each layer consists of randomly generated heterogeneous RFs instead of uniform RFs to encourage diversity and maintain the generality, similar to the method used by gcForest [8]. In this study, a model was designed that uses only the output features of the previous layer as the new input features of the next layer without combining the transformed feature vector. As a replacement for deeper and wider networks, the LMRF model is applied to an embedded system in low-power and low-memory in-vehicle systems for the monitoring of driver emotions. LMRF was also used as an image registration application for real-time keypoints matching in [27].

The deep random ferns (d-RFern) model [28] connects extremely randomized ferns to multiple layers to allow a high classification performance and a lightweight and fast structure. The input vector is first encoded as a transformed feature vector in the feature encoder layer and is then input to the cascade layers. The feature encoding process is similar to the DNN convolution and helps improve the performance of the final output layer. The cascade layer adjusts the number of ferns and layers required for the d-RFern adaptively, using only a small amount of data.

Additional approaches exist in which convolutional neural networks (CNNs) and decision trees [29,30,31] are combined to integrate the DNN architecture with a supervised forest feature detector. However, these differ from ensemble-based approaches that use ensemble trees as a layer-by-layer connection without the use of backpropagation during learning.

Although a deep ensemble classifier-based deep model achieves a good performance similar to that of a DNN, one RF must consist of a few hundred trees, and several RFs must form a single layer. Instead, FTDRF [7] consists of only two layers consisting of 2000 decision trees per layer without the use of several RFs. However, this method also has a disadvantage in that the operation speed is slow owing to an excessive number of trees. In addition, they must be connected to multiple layers and have a similar length and parameter numbers similar to those of a DNN.

### 2.3. Contributions of This Study

Among the numerous deep ensemble models available, in this study, the proposed rule elimination algorithm is applied to the LMRF [24], which is applicable to a real-time system because the numbers of RF neurons and layers are smaller than those of the other methods. This transparent and simplified LMRF (sLMRF) is applied to various datasets to prove that the performance is maintained even when the number of rules is drastically reduced. To design a more transparent and simpler DRF, this paper proposes new contributions in the following order.

**Figure 1:**
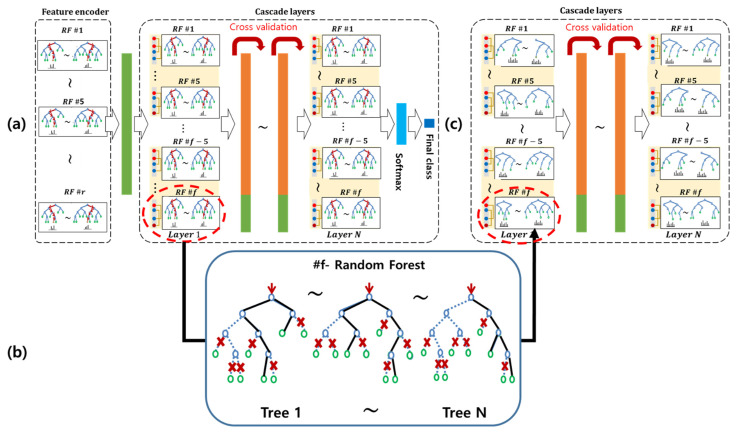
Overall architecture of model simplification using an sLMRF: (**a**) after an LMRF is generated, each RF consists of a large number of decision trees, (**b**) an RF can be simplified by decomposing rules based on the feature contribution, and (**c**) the simplified cascade layers of sLMRF.

- After an LMRF is trained and multi-layer networks are generated using several RFs (see, Figure 1a), we first decompose the predictions of each decision tree in the RF into mathematically exact feature contributions.
- Individual predictions of the decision tree can be explained by breaking down the decision path into a single component per feature. This procedure is iteratively applied to find all rules of the entire RF layer by layer and saved to decision sets, which are sets of classification rules of an RF, (see the example in Figure 1).
- Sequential covering then repeatedly maintains and eliminates rules of the decision set of an RF based on a combination of the rule contribution and feature pattern (frequency of rules). This regularization keeps only a small number of refined rules that are the most discriminative.
- After the sequential covering, we have the same number of decision sets per layer, but the numbers of rules and features are significantly reduced without decreasing the performance.
- Herein, we provide the qualitative and quantitative results demonstrating that our proposed model simplification method is understandable and effective for real-time processing.

## 3. Simplification of Deep Random Forest

In this section, we introduce procedures for building an LMRF and decomposing it into a simplified model, i.e., an sLMRF. Simplification is achieved through an elimination of weak rules based on an analysis of the feature contributions. The primary contribution of this study is to make the sLMRF transparent/simple by creating a new contribution metric for interpreting the classifiers based on the feature contribution and frequency. This process is conducted from the second cascading layer except for the first feature encoding layer in the network, as shown in Figure 1.

We demonstrate herein how the decision-making processes of sLMRF consisting of a black box structure can be made explicable through two processes, namely an estimation of the feature contribution and an elimination of unimportant rules.

### 3.1. Growth Phase: Training of DRF

As the first step, a non-NN style deep model, LMRF, based on an ensemble of RFs is trained. The LMRF consists of multiple layers $L^{l}\;\left(l\in \left\{1,\ldots ,N\right\}\right)$ of RFs $F_{v}^{l}\;\left(\nu \in \left\{1,\ldots ,V\right\}\right)$, as depicted in Figure 1a, where each *v*-th RF of *l*-th layer, $F_{v}^{l}$ consists of numerous decision trees t. In the first layer, the input vector is encoded as a transformed feature vector $\Phi^{l}$ by combining the class probabilities of an individual RF, $\Phi^{1}=[\;P(\Phi_{1}^{1}|F_{1}^{1}),P(\Phi_{2}^{1}|F_{2}^{1}),\ldots ,P(\Phi_{V}^{1}|F_{V}^{1})]$. From the second layer, each layer $L^{l}(l>1)$ is trained using the encoded feature vector of layer l−1 layer, and is also used to generate a new feature vector $\Phi^{l}$ for the next layer or to predict the final class at the final layer. With the LMRF, each neuron of a DNN layer is replaced with the RF, and each layer consists of several types of RFs. Each layer consists of randomly generated heterogeneous RFs instead of uniform RFs to encourage diversity and maintain the generality [24]. To determine whether to expand a layer, LMRF uses a K-fold cross-validation to automatically determine the numbers of layers and parameters while reducing the risk of an overfitting. When the LMRF is converged through a K-fold cross-validation, the final class probability is determined by averaging the class probabilities $\hat{\Phi }$ predicted from each RF, and predicting the final class label with the highest probability.

### 3.2. Sequential Covering Based on Rule Contribution

Sequential covering is a common rule induction procedure that iteratively learns a single rule individually to create a decision set that includes the entire dataset [32]. After a densely coupled black box LMRF model is constructed, the rules of an individual RF should be iteratively saved in a decision set based on a sequential covering procedure.

The basic unit of an LMRF, i.e., a decision tree, is regarded as a rule-based model because the decision procedures that determine the final value depend on if–then conditions represented by the trained node. Each path from the root of the tree to a leaf is a rule that classifies a set of examples. When an instance ${Χ}_{n}$ and its label $Y_{n}$ (part of dataset $D=\left\{\left(X_{1},Y_{1}\right),\ldots ,\left(X_{N},Y_{N}\right)\right\})$ falls into a root node, $X_{n}$ will be passed to a right or left child node that satisfies the split function with a threshold for a specific feature determined during the training step. These steps are repeated until the given data reach a leaf node that creates an optimal feature space. The node consists of pairs of specific feature indexes, a split function with a threshold, and a class distribution, and the chain of overall nodes (decision path or rule) per decision tree is stored in the decision set.

In this study, we modified the sequential covering algorithm to select the optimal rules from each tree and RF. In the classification problem, the feature contribution (importance) represents changes in the feature-specific distribution when instances are split up for a particular feature. To calculate the feature contribution, a decision tree traverses downward until it reaches a leaf. At every specific split, the feature contribution of the feature variable that determines the split is defined as the difference in class probability between a parent and child node. To obtain the final rule contribution, we follow the path from the root node to the leaf node of the data instance and sum all feature contributions of each node. This algorithm extracts the paths (rules) sequentially by looking for the best rule that has a high contribution score.

The rule contribution for the *i*-th rule $r_{i}^{t}$ consisting of D depth on a *t*-th decision is then calculated as follows:

(1) $$r_{i}^{t}=\frac{\sum_{j}^{D}feat.contrib\left(i,j\right)}{\sum^{\;}\#\;class\;of\;a\;tree}$$

where the rule contribution can then be normalized to a value of between zero and one by dividing by the sum of number of classes of the *t*-th tree. The feature contribution of the *j*-th node feat.contrib(i,j) of the *i*-th rule is calculated using the difference in class probability $Pr^{i}=\left\{pr_{1}\ldots pr_{\#class}\right\}$ between a parent (j−1) and child (j) node.

(2) $$feat.contrib\left(i,j\right)=||Pr^{i}\left(j-1\right)-Pr^{i}\left(j\right)||$$

A large positive or negative value of $r_{i}$ means that a rule consisting of several features contributes strongly to the decision class. By contrast, a small positive or negative value of $r_{i}$ means that a rule contributes weakly to the decision class. Values of zero in a contribution means that the feature does not contribute to the decision-making process.

A pre-mining of the feature pattern inspired by [19] is also used for the weight of the rule contribution. A feature pattern is the frequent occurrence of feature values (e.g., x = A). We extract frequently occurring feature patterns from all rules in a decision set dSet(v,l) of the v-th RF and l-th layer. The frequency of a feature pattern is measured based on its support in the decision set:

(3) $$fre_{v}^{l}\;\left(x_{j}=A\right)=\frac{1}{\left|\mathrm{dSet}\left(v,l\right)\right|}\;\;\underset{i\epsilon \mathrm{dSet}\left(v,l\right)}{\sum }I\left(x_{j}^{\left(k\right)}=A\right)$$

where |dSet| is the cardinality of features in $\mathrm{dSet}\left(v,l\right),\;fre\left(x_{j}=A\right)$ quantifies the frequency of feature patterns in the rules of dSet(v,l), and *I* is an indicator function that returns a value of one if the feature $x_{j}$ of the instance *k* is of level A; otherwise, a value of zero is returned. The feature pattern is a normalization of the number of overlapping features among all features included in dSet(v,l).

At the RF level, the final rule contribution $r_{i}^{*}$ in a decision set dSet(v,l) is estimated through a weighted combination of the feature contribution and feature pattern.

(4) $$r_{i}^{*}=r_{i}\cdot \underset{j\epsilon r_{i}}{\sum }fre_{v}^{l}\left(x_{j}\right)$$

In the equation, if the feature patterns included in each rule $r_{i}$ have high frequencies, the final rule contribution $r_{i}^{*}$ increases in proportion to the feature contribution. The rules indSet(v,l) for each RF, $F_{v}^{l}$, are sorted and rearranged in ascending order according to the final rule contribution.

This procedure can be iterated until we extract all rules that cover the RFs of the l-th layer. Table 1 shows the reordering of learned rules in a decision set. The initial rules consist of the feature rule of the **IF** clause and the class probability pairs of the **THEN** clause. However, through the proposed sequential covering process, the rules are reordered according to the final contribution of each rule.

### 3.3. Rule Elimination Phase: Simplifying LMRF

We employed a feature contribution with a feature pattern for a rule contribution to represent the correlation between a trained feature and changes in the class probability. This approach helps with understanding which features, rules, and RFs affect the prediction results of an LMRF. However, an LMRF generates a large number of rules because it is also composed of several black-box RFs. Therefore, the decision-making processes of an LMRF can be made explicable through the elimination process of unimportant rules.

Weak rules in dSet(v,l) are eliminated according to the given final rule contribution $r_{i}^{*}$ and only the rules with a high contribution value remain. Rules included in ${\mathrm{List}}_{\mathrm{dSet}}$ [*l*] can be removed at the same rate for each RF according to the user input, or it can be adjusted for each RF depending on the required accuracy.

Algorithm 1 shows the overall rule elimination procedures based on the feature contribution and patterns for constructing a transparent sLMRF. After completing the training of the sLMRF, test data are input into the first feature encoder layer. The outputs of the first layer are concatenated, and these transformed feature vectors, augmented with the class vector generated by the first layer, are input into the list $\mathrm{dSet}\left({\mathrm{List}}_{\mathrm{dSet}}\right)$ of the l-th layer until the data are mapped to the final layer. The final layer averages the probability values of each class and determines the class with the highest probability value as the final class.
**Algorithm 1:** Rule elimination based on feature contribution and feature pattern1: **Input:** The number of layers N, the number of RFs V, the number of trees *T*, random forest RF, list of dSets ${\mathrm{List}}_{\mathrm{dSet}}$
2: Start with an empty list of dSets 3: ${\mathrm{List}}_{\mathrm{dSet}}=\emptyset$ 4: Learn LMRF 5: **For** each l layer: 6: **For** each v RF: 7: **For** each t tree: 8: - Split a i-th rule from a decision tree 9: - Calculate feature contribution of a i-th rule feat.contrib(i,*), Equation (2) 10: - Calculate rule contribution for i-th rule $r_{i}^{t},\;\mathrm{Equation}\;\left(1\right)$
11: - Add rule and its $r_{i}^{t}$ to dSet(v,l) 12: **End** 13: - Compute feature pattern $fre_{v}^{l}\left(x_{j}=A\right)$ by splitting rules in dSet(v,l), Equation (3) 14: - Re-compute a new rule contribution ${r^{*}}_{i}$, Equation (4) 15: - Sort rules in dSet(v,l) according to ${r^{*}}_{i}$ 16: - Add dSet(v,l) to ${\mathrm{List}}_{\mathrm{dSet}}$ of l-th layer 17:             
${\mathrm{List}}_{\mathrm{dSet}}\left[l\right]={\mathrm{List}}_{\mathrm{dSet}}\left[l\right]+\mathrm{dSet}\left(v\right)$ 18: **End** 19: **End** 20: **Output:** The ${\mathrm{List}}_{\mathrm{dSet}}\left[l\right]$ consists of l layers

## 4. Experimental Results

In this section, we check the simplification of the sLMRF model and compare the performance when the same rule elimination is applied to other DRF-based methods and DNN-based compression approaches. From the experiments, we prove that the compressed sLMRF maintains a similar performance, not only the original LMRF, but also DNN-based algorithms, although the sLMRF removes a significant percentage of the rules. To prove the coherence of the performance without overfitting and examine the transparency of the compressed sLMRF, we conduct a test using various kinds of datasets such as facial emotion images, handwritten data, face images, tabular pattern data, and medical diagnosis data.

### 4.1. Datasets

**CK+ dataset** [33]: The expanded Cohn–Kanade (CK+) dataset is a public benchmark dataset for facial expression recognition (FER) and has 327 image sequences from 118 subjects and facial expression labels based on FACS. All sequences start with a neutral state and proceed to a peak state. During the experiments, we use the six facial expression classes of angry, disgust, fear, happiness, sadness, and surprise. The feature vector consists of 84 dimensional distance ratios and 88 dimensional angles that are extracted from the facial landmarks, instead of a feature encoder layer.

**MNIST dataset** [34]: The Modified National Institute of Standards and Technology (MNIST) dataset contains images of handwritten digits and is also widely used for an evaluation in the field of machine learning. The pixels of the images are in black and white, and the images were normalized to a 28-pixel × 28-pixel resolution with grayscale values. The MNIST dataset includes 60,000 training samples and 10,000 testing samples.

**WDBC dataset** [35]: The Wisconsin Diagnostic Breast Cancer (WDBC) dataset provides the diagnosis results of the Wisconsin University Hospital. It is composed of two category labels, malignant and benign, with 212 and 357 images, respectively. The feature vectors of the WDBC dataset consist of 32 variables, including the patient id, diagnosis, radius, texture, perimeter, area, smoothness, compactness, concavity, and symmetry. The feature vectors are used as input vectors for the sLMRF and the comparison methods without a feature extraction encoder.

**ORL dataset** [36]: The Orivetti Research Lab (ORL) dataset contains a set of facial images taken at AT&T Laboratories Cambridge. It offers 400 grayscale images with a pixel resolution of 64 × 64 captured from 40 distinct subjects. Some images were taken at different times, with varying lighting and facial emotions (open/closed eyes, smiling/not smiling) and facial details (glasses/no glasses). All images were captured against a dark background at a frontal position. The 4096 feature vectors from the 64-pixel × 64-pixel resolution are inputted into a feature encoder layer during the experiments on the DRF method.

**Figure 2:**
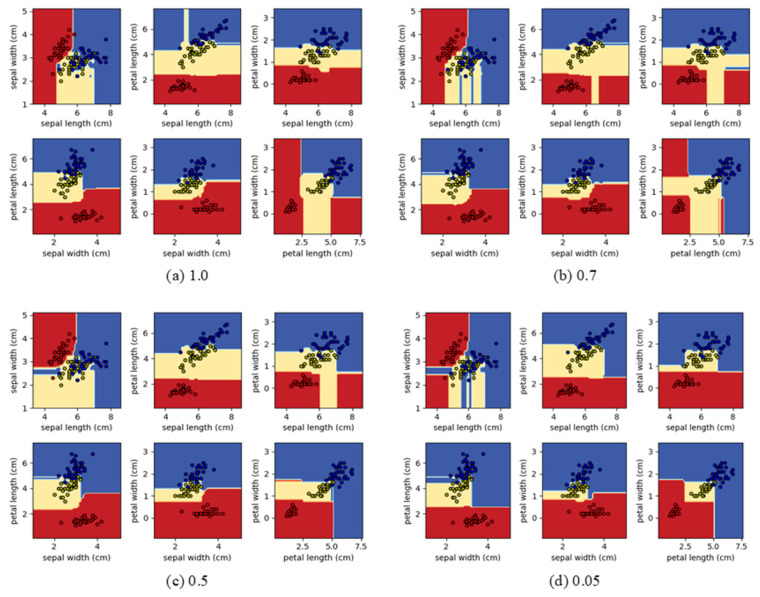
The visualization of decision boundary changes according to the ratio of rule elimination of the fully trained sLMRF using IRIS dataset. The boundary is more obvious in the color image: (**a**) rule ratio 1.0 (**b**) rule ratio 0.7 (**c**) rule ratio 0.5 and (**d**) rule ratio 0.05.

**IRIS dataset** [37]: The IRIS dataset includes three iris species (setosa, versicolor, and virginica) and four-dimensional feature vectors (sepal length, sepal width, petal length, and petal width) and consists of 150 samples. During the experiments, DRF models utilize the four-dimensional feature vectors without the feature encoder layer (first layer) for the feature extraction. The IRIS dataset was used for visualization of decision boundary changes according to the ratio of rule elimination (Figure 2) instead of evaluation of the DRF model because the number of samples and the dimension of the feature vector are small.

### 4.2. Evaluation of DRF Models

One way to prove the transparency of a model is to show its simplicity. Therefore, during this experiment, we first compared the numbers of rules, parameters, and operations used in the model, and the accuracy, while reducing the model size for the DRF-based methods. To verify the effectiveness of the rule elimination scheme, we compared its performance with that of two representative DRF-based methods by varying the ratios of the rules from 1.0 to 0.6: (1) sLMRF, (2) gcForest [8], and (3) FTDRF [7]. Table 2 and Table 3 demonstrate the performance according to the rule ratios using the CK+ and MNIST datasets, respectively.

As we can see from Table 2, when sLMRF is trained using fully connected rules with CK+ facial landmark features, the accuracy is somewhat higher than that of gcForest (3.89%) and FTDRF (1.19%) despite using a slightly smaller number of trees (rules) and RFs. The numbers of parameters and operations of sLMRF are also 5.5 and 6.4 times lower than those of gcForest, and 1.9 and 3.9 times lower than those of FTDRF, respectively. However, when we reduced the rule ratio by 40% (0.6), the accuracy of sLMRF decreased by 2.1% compared with the fully connected rules, although the relative accuracy is still higher than that of gcForest and FTDRF. As sLMRF originally used fewer rules, the more rules that are removed, the lower the performance compared to the other methods. However, the required numbers of parameters and operations for a compressed sLMRF are 5.4 and 6.4 times smaller than those of gcForest, and 4.6 and 3.9times smaller than those of FTDRF.

As shown in Table 3, the three algorithms used a feature encoder layer to transform the MNIST images into a new input vector. The original sLMRF using fully connected rules has a slightly lower accuracy than that of gcForest and FTDRF because it uses a smaller number of trees (rules) and RFs. For example, gcForest and FTDRF use 3.25 and 11.8 times more rules than sLMRF, respectively. In addition, sLMRF also has 12.5 and 9.6 times fewer parameters and operations than gcForest, and 3.4 and 3.3 times fewer parameters and operations than FTDRF. When we reduce the rule ratio by 40% (0.6), the accuracy of sLMRF is only 1.9% lower than that of gcForest and FTDRF. However, the number of rules learned by sLMRF is approximately 11 times lower than that of gcForest and 3 times lower than that of FTDRF. In addition, the numbers of parameters and operations of sLMRF are 12.3 and 9.7 times lower than those of gcForest, and 3.4 and 3.4 times lower than those of FTDRF, respectively. From the results, we can see that the proposed rule elimination effectively reduces the number of parameters and operations, and a finer gap in the accuracy of sLMRF may be sufficiently acceptable for mobile devices as well as real-time embedded systems.

To evaluate the performance of the algorithm on more diverse datasets, we conducted the same experiment on the WDBC, and ORL datasets. As shown in Table 4 and Table 5, although sLMRF uses much fewer parameters and operations, it demonstrates a similar accuracy as gcForest and FTDRF. Based on the experiment results, we confirmed that sLMRF achieves an efficient rule compression among DRF models in terms of both memory and the number of computations. Exceptionally, our approach has slightly less maintainability in terms of accuracy than gcForest and FTDRF according to the changing rule elimination ratio for the ORL dataset. The reason for this is that the original sLMRF consists of small networks with only a few core rules, although gcForest and FTDRF models have a higher rule redundancy in the network. Therefore, although the rules of the two comparison methods are reduced, most of the duplicated rules are removed, and thus the performance is not significantly reduced.

Overall, although the three methods commonly remove numerous rules compared to their original model, gcForest and FTDRF still contain larger rules from a minimum of 1.6 (gcForest of ORL) to a maximum of 3.8 times (FTDRF of WBC) those of sLMRF when rule ratio is 0.6, although the accuracies remain similar.

### 4.3. Decision Boundary Analysis

To visualize the effect of rule interpretation on sLMRF based on contribution-based rule elimination, the analysis results using IRIS dataset are shown in Figure 2. As represented in Figure 2, although the rule elimination rate is changed from fully trained sLMRF for each feature pair (sepal length, sepal width, petal length, and petal width), the decision boundary is formed similar to the original sLMRF without overfitting to specific data.

### 4.4. Comparison with State-Of-The-Art Methods

An additional experiment was conducted on the CK+ dataset to test whether the proposed algorithm effectively recognizes the facial expressions, and the performance was compared with other state-of-the-art-methods, namely an AlexNets-based FER approach [38]; a 3D CNN-based FER approach with deformable facial action parts constrained (3DCNN-DAP) [39]; a DNN-based approach that uses multiple inception layers [40]; a 3D Inception-ResNet (3DIR) with LSTM for the FER [41]; three DRF-based methods, i.e., gcForest [8], FTDRF [7], and LMRF [24]; and the proposed sLMRF. The DRF-based methods, gcForest, FTDRF, and sLMRF, exploited a feature vector consisting of an 84-dimensional distance ratio and an 88-dimensional angle ratio [42] without using the entire image.

**Figure 3:**
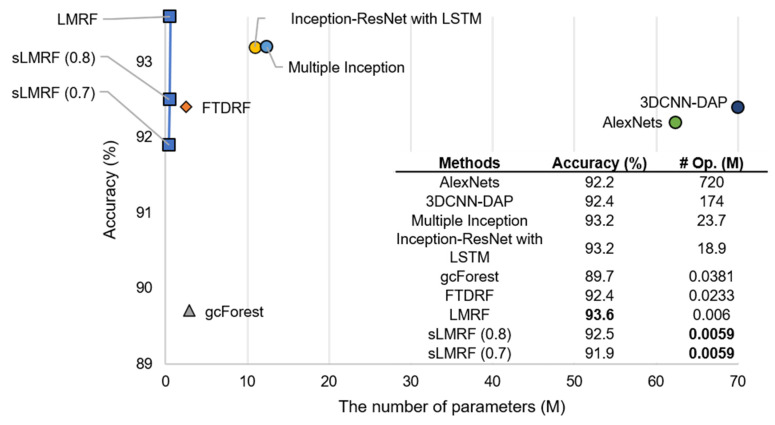
Comparison of accuracy, numbers of parameters (# Param.), and numbers of operations (#Op.) with the state-of-the-art methods using the CK+ dataset.

As shown in Figure 3, although 30% of the rules are removed through a rule elimination from the original sLMRF model, the resulting accuracy is only 1.3% less than that of the approaches described in [40] and [41]. However, the overall numbers of parameters and operations are significantly reduced compared to the two DNN-based algorithms.

In the second comparison, we conducted a test on the MNIST dataset and compared the performance between the three state-of-the-art CNN-based methods and two DRF methods, namely, ResNet-101 [43] and three CNN compression networks, shuffleNetV2 [44], MovileNetV2 [45], and MovileNetV3 [46]; DRF-based methods, i.e., gcForest [8], FTDRF [7], and LMRF [24]; and the proposed sLMRF.

**Figure 4:**
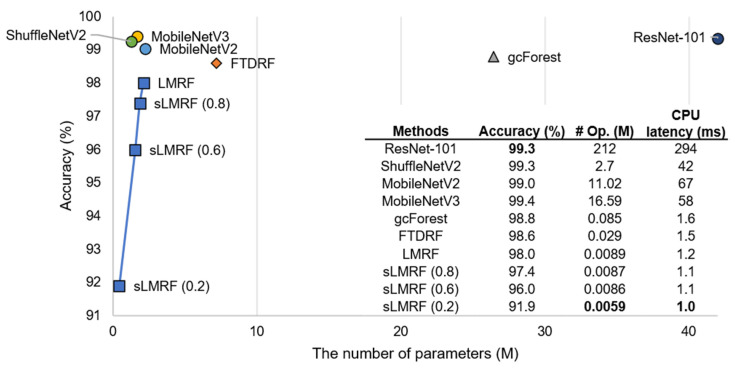
Comparison of accuracy, numbers of parameters (# Param.), numbers of operations (#Op.) and CPU latency with the state-of-the-art methods using the MNIST dataset.

Figure 4 shows that the accuracy of the original sLMRF is similar to that of the state-of-the-art methods. When 40% of the rules of the sLMRF are removed, only a 2% decrease in the existing accuracy occurs. The reduced accuracy can be overcome when considering the effectiveness of the numbers of parameters and operations of the sLMRF when compared against other CNN-based compression algorithms [43,44,45,46]. For example, in the case of MobileNetV3, the accuracy is 3.4% higher than that of sLMRF (0.6), although the number of operations of sLMRF is 1929 times higher. In addition, we evaluated inference latency on the CPU for the MNIST dataset. We referenced off-the-shelf CNN-based methods [43,44,45,46] and DRF-based methods [7,8]. As illustrated in Figure 4, DRF-based methods outweigh the CNN-based methods, specifically, the sLMRF shows gradually decreased results as following the rule downsizing. ShuffleNetV2 which is the fastest method among CNN-based methods takes 42 times more latency than the sLMRF (0.2).

Through the two experiments, we know that the proposed method can derive outstanding compression performances in terms of the numbers of parameters and operations while maintaining the level of accuracy and will be an opportunity to extend the range of sLMRF applications to low-end systems.

## 5. Conclusions

In this paper, a transparency and simplification method for a black box DRF model using rule elimination based on the feature contribution and pattern was proposed. The model simplification is achieved by analyzing the importance of the features on the sub-optimal space of each node from a fully trained sLMRF and eliminating the low contribution rules. Although DNN-based model compression methods should consider the trade-off regarding the number of parameters and the performance, the experimental results proved that the proposed method effectively reduces the number of rules, parameters, and operations without a decrease in performance. In addition, unlike a black-box model, we can interpret which features contribute most to the decision-making of the sLMRF before making the final decision through the rule elimination process. However, the proposed sLMRF method is not a fully white model because it still contains numerous rules and feature parameters. In addition, sLMRF has excellent classification performance for tabular data obtained from sensors, but the performance of extracting feature vectors by encoding continuous data such as video and audio is still inferior to that of CNN.

A future study will focus on the design of a fully interpretable model that is human understandable through a depth-wise analysis of the rules. Moreover, current algorithms need to increase the number of RFs of the feature encoder layer to process large-sized image like ImageNet but will develop additional encoder that can extract efficient image features with fixed RFs.

## Figures and Tables

**Table 1:** One example of decision set reordering. The first five rules are extracted from the *v*-th RF and are rearranged based on the final contribution. Each rule has a pair of contributions and probabilities of a class.

Rules
**Initial Rules of**dSet(v,l) $Rule\;1:\;\left(x_{2}>2.59\right)$ and $\;\left(x_{2}>4.75\right)$ and $\left(x_{0}<=6.04\right)$ and $\left(x_{3}>1.84\right)$ ⇒ {0} [0, 0, 1] $Rule\;2:\;\left(x_{2}>2.59\right)$ and $\;\left(x_{2}>4.75\right)$ and $\left(x_{0}<=6.04\right)$ and $\left(x_{3}<=1.84\right)$ ⇒ {-0.277} [0, 0.5, 0.5] $Rule\;3:\;\left(x_{3}<=1.75\right)$ and $\;\left(x_{3}>0.7\right)$ and $\left(x_{3}<=1.55\right)$ ⇒ {0.55} [0, 1, 0] $Rule\;4:\;\left(x_{3}<=1.75\right)$ and $\;\left(x_{3}>0.7\right)$ and $\left(x_{3}>1.55\right)$ ⇒ {0.27} [0, 0.75, 0.25] $Rule\;5:\;\left(x_{3}>0.7\right)$ and $\;\left(x_{3}>1.55\right)$ and $\left(x_{2}<=4.95\right)$ ⇒ {0.83} [0, 0.6, 0.4] ……
**Reordered Rules of**dSet(v,l) $Rule\;1:\;\left(x_{3}>0.7\right)$ and $\;\left(x_{3}>1.55\right)$ and $\left(x_{2}<=4.95\right)$ ⇒ {0.83} [0, 0.6, 0.4] $Rule\;2:\;\left(x_{3}<=1.75\right)$ and $\;\left(x_{3}>0.7\right)$ and $\left(x_{3}<=1.55\right)$ ⇒ {0.55} [0, 1, 0] $Rule\;3:\;\left(x_{2}>2.59\right)$ and $\;\left(x_{2}>4.75\right)$ and $\left(x_{0}<=6.04\right)$ and $\left(x_{3}<=1.84\right)$ ⇒ {-0.277} [0, 0.5, 0.5] $Rule\;4:\;\left(x_{3}<=1.75\right)$ and $\;\left(x_{3}>0.7\right)$ and $\left(x_{3}>1.55\right)$ ⇒ {0.27} [0, 0.75, 0.25] $Rule\;5:\;\left(x_{2}>2.59\right)$ and $\;\left(x_{2}>4.75\right)$ and $\left(x_{0}<=6.04\right)$ and $\left(x_{3}>1.84\right)$ ⇒ {0} [0, 0, 1] ……

**Table 2:** Comparison of accuracy, number of rules, number of parameters (#Param.), and number of operations (#Op.) between DRF models according to the rule ratios using the CK+ dataset. The rule ratio 1.0 of sLMRF is the same as that of LMRF.

Rule Ratio	Accuracy (%)			Rules (M)			# Param. (M)			# Op. (M)		
	sLMRF	gcForest	FTDRF	sLMRF	gcForest	FTDRF	sLMRF	gcForest	FTDRF	sLMRF	gcForest	FTDRF
1.0	**93.60**	89.71	92.41	**0.12**	0.16	0.13	**0.53**	2.90	2.51	**0.0060**	0.0381	0.0233
0.9	**92.86**	90.00	92.15	**0.11**	0.15	0.12	**0.51**	2.78	2.39	**0.0060**	0.0381	0.0232
0.8	**92.50**	89.92	92.24	**0.09**	0.13	0.10	**0.47**	2.59	2.22	**0.0059**	0.0380	0.0231
0.7	91.87	89.92	**92.18**	**0.08**	0.12	0.09	**0.44**	2.38	2.03	**0.0059**	0.0379	0.0230
0.6	91.05	89.73	**92.04**	**0.07**	0.10	0.08	**0.39**	2.16	1.83	**0.0058**	0.0377	0.0228

**Table 3:** Comparison of accuracy, number of rules, number of parameters (#Param.), and number of operations (#Op.) between DRF models according to the rule ratios using the MNIST dataset. The rule ratio 1.0 of sLMRF is the same as that of LMRF.

Rule Ratio	Accuracy (%)			Rules (M)			# Param. (M)			# Op. (M)		
	sLMRF	gcForest	FTDRF	sLMRF	gcForest	FTDRF	sLMRF	gcForest	FTDRF	sLMRF	gcForest	FTDRF
1.0	97.98	**98.77**	98.57	**0.08**	0.94	0.26	**2.12**	26.42	7.17	**0.0089**	0.0852	0.0296
0.9	97.77	**98.73**	98.57	**0.08**	0.85	0.23	**2.00**	24.79	6.76	**0.0088**	0.0851	0.0294
0.8	97.41	**98.74**	98.57	**0.07**	0.76	0.21	**1.86**	22.98	6.28	**0.0087**	0.0850	0.0293
0.7	96.86	**98.76**	98.47	**0.06**	0.66	0.18	**1.71**	20.98	5.75	**0.0087**	0.0849	0.0292
0.6	96.00	**98.75**	98.39	**0.05**	0.57	0.16	**1.54**	18.86	5.19	**0.0086**	0.0850	0.0291

**Table 4:** Comparison of accuracy and numbers of rules among three DRF models according to changes in rule elimination using the WDBC dataset. The rule ratio 1.0 of sLMRF is the same as that of LMRF.

Rule Ratio	Accuracy (%)			Rules (M)		
	sLMRF	gcForest	FTDRF	sLMRF	gcForest	FTDRF
1.0	96.49	95.21	**97.34**	**0.0063**	0.0450	0.0256
0.9	96.49	95.21	**97.34**	**0.0063**	0.0450	0.0247
0.8	96.49	95.21	**97.34**	**0.0057**	0.0385	0.0227
0.7	96.49	95.74	**97.34**	**0.0050**	0.0350	0.0199
0.6	96.49	95.74	**96.81**	**0.0044**	0.0298	0.0168

**Table 5:** Comparison of accuracy and numbers of rules among three DRF models according to changes in rule elimination using the ORL dataset. The rule ratio 1.0 of sLMRF is the same as that of LMRF.

Rule Ratio	Accuracy (%)			Rules (M)		
	sLMRF	gcForest	FTDRF	sLMRF	gcForest	FTDRF
1.0	**97.50**	**97.50**	90.00	**0.0595**	0.0957	0.1133
0.9	**97.50**	**97.50**	90.00	**0.0543**	0.0893	0.1045
0.8	**97.50**	**97.50**	90.00	**0.0481**	0.0786	0.0925
0.7	87.50	**97.50**	90.00	**0.0422**	0.0702	0.0809
0.6	87.50	**97.50**	90.00	**0.0362**	0.0594	0.0696

## Data Availability

Publicly available datasets were analyzed in this study. These data can be found here: [https://www.kaggle.com/c/visum-facial-expression-analysis, http://yann.lecun.com/exdb/mnist/, https://datahub.io/machine-learning/wdbc, https://cam-orl.co.uk/facedatabase.html, https://archive.ics.uci.edu/ml/datasets/iris (accessed on 20 April 2021)].
